# Potential impact of extra education on the development of executive functions within a year in preschool children: an exploratory research

**DOI:** 10.3389/fpsyg.2023.1193472

**Published:** 2023-05-23

**Authors:** Alexandra Dolgikh, Larisa Bayanova, Elena Chichinina

**Affiliations:** ^1^Laboratory of Childhood Psychology and Digital Socialization, Psychological Institute, Russian Academy of Education, Moscow, Russia; ^2^Department of Educational Psychology and Pedagogy, Faculty of Psychology, Lomonosov Moscow State University, Moscow, Russia

**Keywords:** executive functions, inhibitory control, working memory, cognitive flexibility, preschool age, children, extra preschool education

## Abstract

Executive functions have been shown to develop through various extra classes in preschool age. But the optimal for executive functions development system of such classes has not yet been explored. The present exploratory study aimed to examine the difference in the executive functions development within a year between children attending the system of extra classes (music, choreography, art, foreign language, literacy, math, computer science, and science) twice a week for 4 h in a preschool education center and children who did not take no extra classes. There were 60 children who attended extra classes and 64 children who did not take extra classes. In each group, approximately 17% were boys. The first assessment of executive functions was performed in the penultimate year of kindergarten, when the children were 5–6 years old. The second was performed 1 year later. The executive function level was assessed using NEPSY-II subtests “Inhibition,” “Statue,” “Memory for Designs,” “Sentences Repetition,” and “Dimensional Change Card Sort.” Mothers also reported about their children’s attendance in extra classes, their children’s screen time, the level of maternal education, and the level of family income. The study revealed that children attending the system of the extra classes showed a higher verbal working memory development within a year than the children taking no extra classes. The obtained data plays an important role for the design of further research of the topic and for the practical recommendations for parents and teachers.

## 1. Introduction

Executive Functions (EF) is an umbrella term for cognitive processes that underpins goal-directed behaviors, cognitive processes, and adaptive behavior in new situations (Diamond, 2013; Friedman and Miyake, 2017). EF can be divided into three interrelated components: inhibitory control (cognitive and physical), working memory (verbal and visual), and cognitive flexibility (Miyake et al., 2000). A large number of empirical results reveal that the level of EF in preschool age is a predictor of school readiness, academic performance in school (Robson et al., 2020; Kovyazina et al., 2021; Morosanova et al., 2021), and social competence (Denham et al., 2010; Shen et al., 2020). Furthermore, the level of EF development in children is associated with physical and mental health, and social competence in adulthood (Moffitt et al., 2011; Stichter et al., 2016). A low EF level in childhood is associated with internalizing and externalizing problems, antisocial behavior, health problems, aggression, peer victimization, and depression (Robson et al., 2020). Therefore, EF are a necessary foundation for full-fledged cognitive and socio-emotional development in both childhood and adulthood (Scionti et al., 2020).

Due to the great importance of the EF in childhood, researchers study how to influence the EF development. The quality of parent–child interaction and parenting methods play an important role in promoting the development of EF in childhood. Therefore, the level of EF in children is positively related to parenting behaviors such as warmth, responsiveness, autonomy, support, scaffolding, and negatively related to parents’ intrusiveness and detachment (Valcan et al., 2018).

One of the most effective ways to develop the EF of preschoolers is a game (Yogman et al., 2018). The effectiveness of this method is derived from the fact that the game is the leading activity in this age (Vygotsky, 2012). The game develops the ability to follow instructions, problem-solving skills, working memory, cognitive flexibility, and emotional, cognitive, and physical inhibitory control (Yogman et al., 2018). The game harmonizes the emotional state of the child and, in turn, creates favorable conditions for the EF development (Rosas et al., 2019).

Additionally, screen time matters when considering the EF development. It has been proven that excessive screen time is associated with a lower level of EF in preschoolers (Linebarger et al., 2014; McNeill et al., 2019). The truth is that excessive screen time can replace parent–child interaction, live play, physical activity, and the necessary amount of sleep. Consequently, it has a negative impact on the EF.

Sleep is also closely related to the development of EF. Children who do not sleep enough may have a lower level of EF (Kahn et al., 2021). Another important factor that can influence EF development is physical activity (Bai et al., 2022). So, preschoolers are recommended to engage in at least 180 min of physical activity per day to ensure normal cognitive development (McNeill et al., 2020; Veraksa et al., 2021). Physical activity itself is conducive to the EF development because it increases blood flow, general sensorimotor stimulation (van den Berg et al., 2019), and activation of brain structures, primarily the Third Functional Unit (Tvardovskaya et al., 2020), responsible for programming, regulation, and control of mental activity. At the same time, there is evidence that physical activity stimulates the EF development only if it is purposeful and specially organized—for example, if it involves sport training, dancing training, or sports games (Chang et al., 2012; Tomporowski and Pesce, 2019).

Executive function has been shown to develop through various activities in preschool education programs and classes (Rosas et al., 2019). For example, studies have shown that regular attendance of cognitive training (Röthlisberger et al., 2011), dance classes (Shen et al., 2020; Rudd et al., 2021), music classes (Chen et al., 2022), foreign language classes (Frolli et al., 2022), sports (Jarraya et al., 2019; Tvardovskaya et al., 2020), and other activities is associated with the development of EF in preschool children.

Any organized class, be it dancing, music, etc., must meet a number of requirements to contribute to the development of EF. Firstly, the EF development depends on the amount of time spent practicing, the frequency, and regularity of classes (Diamond and Ling, 2016). Secondly, the tasks that are set for the child during the classes should always be in the “zone of proximal development” (Vygotsky, 2012; Diamond and Ling, 2016). EF must always be challenged (Diamond and Ling, 2016). Thirdly, activities should support the child’s motivation and promote enjoyment, self-confidence, and the development of social connections (Rosas et al., 2019). The individual characteristics of the child also play a role. Children with the poorest EF benefit the most from the activities in terms of EF development (Diamond and Ling, 2016).

Children’s various activities attendance has become very popular among residents of developed countries in recent years (Wang et al., 2023). However, the optimal set of classes and the ideal schedule of such classes for the development of the EF have not yet been explored. Moreover, there is no unequivocal data on whether the attendance of extra classes is really associated with the development of the EF. In this regard, a research question was: Will attending extra classes during the year be associated with the children’s EF development in comparison to the ones who do not attend extra classes? The main hypothesis of the research suggested that children attending extra classes had a higher level of EF development within a year compared to children taking no extra classes. The study considered some factors that can affect EF: screen time, mother’s education level, and family income level.

Most studies with preschool children are experiments where children take part in a training of one type of activity (e.g., dance or cognitive training) for a specified period (e.g., 2 months). With this design of the study, all children attend all classes in the cycle and attend only these classes. In addition, classes are conducted by specially trained people who know about the experiment. Thus, these experiments have low ecological validity. The truth is that, in reality, children regularly miss classes, attend different types of classes, and all teachers behave differently during the class. In the present research, the requirements of ecological validity are met. All children in both groups participated in the general educational program offered by kindergartens, as do children in kindergartens in most countries of the world (Veraksa et al., 2019). Also, the children were not specially selected for participation in the study: Those children who already attended the center of extra preschool education were invited to participate, thus, reflected the real characteristics of preschoolers taking extra classes and their families.

This study compares children who do not participate in any extra classes and children who attend an extra preschool education center where classes are held twice a week for 4 h. During these 4 h, children attend a whole set of eight different classes (music, choreography, art, foreign language, literacy, math, computer science, and science) for 20 min. This exploratory study will allow to articulate the recommendations for further research in this direction.

## 2. Methods

### 2.1. Procedure

Executive Function evaluation was performed in the first half of the school year (in the fall). The first EF evaluation (T1) was performed in the penultimate year of kindergarten, when the children were 5–6 years old. The second evaluation (T2) was performed 1 year later in the last year of kindergarten, when the children were 6–7 years old. The level of EF was assets with the same measures in the first (T1) and the second (T2) evaluation. The EF evaluation included two individual meetings with each child (сa. 20 min. Each meeting). The tasks were always given to the children in the same order. EF assessments were carried out by specially trained testers. If for some reason the child did not want to continue the testing procedure, then it was stopped.

In T2, an online survey of mothers was also conducted. Mothers of children participating in T1 received a link to the questionnaire through parental chat on messengers. Completing the questionnaire took about 20 min. Not all mothers who received a link to the questionnaire completed it. Consequently, at this stage, the selection of the children took place based on their mothers’ interest in participating in the study.

Before the start of the study, written informed consent was obtained from the parents. At the end of the study, each parent was individually provided with feedback on the child’s EF evaluation results, and recommendations were given. Additionally, during this meeting, the parent had the opportunity to consult with a psychologist about the development of the child. This meeting was used as a motivation to participate in the study.

The study was approved by the Ethics Committee of the Lomonosov Moscow State University (approval no: 2022/23).

### 2.2. Sample

The sample of this study consisted of 124 typically developing children from Kazan, Krasnodar, Moscow, Perm, and Yakutsk. The mean age of the children in the last year of kindergarten (T2) was 78 months (see Table 1). All children spoke Russian without developmental delays. All children within the framework of the general program of the kindergarten attended classes aimed at cognitive, speech, physical, artistic-esthetic, and socio-communicative development (Veraksa et al., 2019).

**Table 1:** Differences in sex, maternal education level, family income level, age, and screen time between the groups.

		No extra classes, *n* = 64		Extra classes, *n* = 60		Differences	
		%		%		Chi-squared test	*p*-level
Sex	Boys	17.2		18.3		0.024	0.877
	Girls	82.8		81.7			
Maternal education	Secondary general education	12.3		0.0		9.141	0.027
	Secondary vocational education	31.6		11.1			
	Higher education	56.1		83.3			
	Academic degree	0.0		5.6			
Family income	Low	8.6		0.0		2.170	0.338
	Average	77.6		77.8			
	Above average	13.8		22.2			
		M	SD	M	SD	U-Mann–Whitney Test	*p* level
Age, 6–7 years, months		78.37	3.39	78.40	2.77	1694.500	0.806
Screen time at the age 6–7, minutes per week	Total screen time	1298.86	603.86	1016.00	516.02	341.000	0.061

There were two groups of participants: children attending extra preschool education center throughout the year (*n* = 60) and children who did not participate in any extra classes (*n* = 64). In each group, approximately 17% were boys, approximately 80% of the families had a medium level of income (see Table 1). There are statistically significant differences between the groups in the level of maternal education (see Table 1). Therefore, in the group of children who attended the extra preschool education center, more mothers had higher education. The average screen time of children in both groups is about 1,000–1,300 min per week. There are no statistically significant differences in screen time between the groups, but *p*-level is close to significant (see Table 1). In the “no extra classes” group, screen time is about 3 h per day on average (1298.86 min per week), and in the “extra classes” group it is about 2.5 h per day (1016.00 min per week).

#### 2.2.1. “Extra classes” group

These children participate in a training, which is conducted by the extra preschool education. Classes under this training program are held twice a week for 4 h. During the 4 h, children attend various classes, each of which takes place in its classroom and lasts 20 min. The program includes the following classes: music, choreography, art, foreign language, literacy, math, computer science, and science. All of these children are also attending kindergarten and kindergarten classes.

#### 2.2.2. “No extra classes” group

These children, as well as children from the “Extra classes” group, attend classes that are held in kindergarten (Veraksa et al., 2019). All children in kindergartens attend dance, music, physical education, drawing, and science at least twice a week. A reparatory-for-school classes (reading, writing, mathematics, etc.) children attend every day. Apart from kindergarten, these children do not attend any other extra classes.

The formation of these two groups went through several stages. First, at the T1, 180 preschoolers who attended extra preschool education center took part in the EF assessment. A year later, in T2, 72 children out of 180 took part in the reevaluation of the EF. There were also 110 children in the large longitudinal study who did not attend additional classes according to the mother’s questionnaire. These children took part in the EF assessment at the same time (T1 and T2) as children who attended extra preschool education center. For each child attending the extra preschool education center, a child of the same age, sex, and screen time, with the mother of the same education level, and from a family with the same income level was selected from the 110 children who did not take extra classes. In both groups, children with maximum scores in the T1 were excluded from the sample. In the end, there were 64 and 60 children in the respective groups (17% boys). The inequality in sex distribution between the groups is because there were only 11 boys in the “extra classes” group.

### 2.3. Method

#### 2.3.1. Questionnaire for mothers

The questionnaire for mothers asked about their children’s attendance of any extra classes, in addition to those classes that are offered for all as part of the kindergarten program. The questions were about music, drawing, dancing, sports, math, literacy, foreign language, and classes with a neuropsychologist and a speech therapist, and other classes that mothers could indicate themselves. The following questions were asked about each type of classes:

1. What kind of activities does the child attend?
2. How many times a week does the child attend classes?
3. How long does the class last? (minutes).
4. How many years have the child been attending the classes?

In the questionnaire, there were also questions about child’s screen time, maternal education level, and family income level.

#### 2.3.2. Executive functions assessment

NEPSY-II subtest (Korkman et al., 2007) “Inhibition” was used to evaluate cognitive inhibitory control. A series of 40 figures (squares, circles, and arrows) make up this technique. The test consists of two sections: Naming (the child is asked to label the figures with the fastest speed possible) and Inhibition (reverse Naming, that is, when a square is shown, it should be labeled “a circle” and so on). Time devoted to each task, the number of mistakes, and the times of self-adjustments are recorded. These three values are converted into a combined scaled score (from 1 to 20 points) based on corresponding tables. The combined scaled score for “Inhibition” was incorporated in the analysis.

The NEPSY-II subtest (Korkman et al., 2007) “Statue” was used to evaluate physical inhibitory control. During this task, a child has to stay immobile for 75 s without being disturbed by special sound stimulus (knocking, coughing, and a sound of a pen that fell on the floor). For each 5-s period, a child received 0–2 points for successfully following instructions (maximum number of points = 30). Such mistakes as additional movements, eye opening, and various sounds are recorded. The total score was incorporated in the analysis.

Verbal working memory was evaluated with the help of the NEPSY-II subtest (Korkman et al., 2007) “Sentences Repetition” This instrument comprises 17 sentences that progressively become more difficult to remember as the sentences expanded in length and became more grammatically intricate. Participants receive points for correctness: two points are given for each correctly repeated sentence, one point for a sentence with one or two errors, and 0 points for 3 and more errors (maximum points = 34). In this article, the analysis includes only the total score assigned for the correct repetition of sentences.

To assess visual working memory, the NEPSY-II subtest (Korkman et al., 2007) “Memory for Designs” was utilized. Participants are shown a picture with vibrant images in different cells of a field (four trials with four, six, six, and eight images). After the children have 10 s to look at the picture, it is removed and the respondents have to find the exact images of the set and place them in the appropriate cells on a blank field. The children receive two points for each correctly selected card (picking a similar card gave a child one point) and one point for accurately selecting the position of the card on the field. Moreover, if the participant places the correct card in its proper location, he or she receives two bonus points. Subsequently, the content score (maximum points = 48), location score (maximum points = 24), bonus score (maximum points = 48), and the total score (maximum points = 120) are calculated. Only the content score was examined in the article.

“Dimensional Change Card Sort” task (Zelazo, 2006) was utilized to evaluate cognitive flexibility. Three task sequences that incorporate sorting cards with the images of rabbits and boats using different rules are included in this tool. The initial sequence requires child to separate six cards by color (put red cards to one side, blue ones to the other). The following sequence requires the child to separate six cards by shape (boats and rabbits separately). In the final activity, participants are expected to be guided by a stimulus not related to color or shape (presence or absence of a black frame on the picture). Children should separate the 12 cards according to the shape or color of the object and the frame. Participants gain one point for each correctly sorted card, and those points are combined into the total score (maximum points = 24). In this article, the total score was used.

## 3. Results

The descriptive statistics for the measures of the executive function are presented in Table 2. In the present study, we calculated development rate of EF within a year (Δ), where Δ = EF level at 6–7 years (T1) − EF level at 5–6 years (T2). Nonparametric Mann–Whitney U-test for independent samples was used onwards because not all parameters were distributed normally. According to the Mann–Whitney U test, there is a difference between groups in verbal working memory development rate. So, children attending the extra preschool education center showed a higher verbal working memory development within a year than the children taking no extra classes (see Table 3). There are also statistically significant differences between the groups in the level of verbal working memory in T1 and T2 (see Table 3). There are no other statistically significant differences between the groups in the rates of EF development (see Table 3).

**Table 2:** Descriptive statistics for executive functions measures.

	Group	*n*	Min.	Max.	Median	Mean	SD	Skewness	SD	Kurtosis	SD
Δ Inhibition	No extra classes	54	−10	8	1.00	0.574	0.569	−0.650	0.325	0.335	0.639
	Extra classes	55	−9	6	0.00	0.218	0.421	−0.936	0.322	1.13	0.634
Δ Physical Inhibition	No extra classes	46	−16	8	0.00	0.391	0.582	−1.52	0.350	6.19	0.688
	Extra classes	42	−10	6	0.00	−0.429	0.588	−0.625	0.365	−0.0175	0.717
Δ Verbal working memory	No extra classes	62	−7	18	1.00	1.39	0.562	0.867	0.304	2.18	0.599
	Extra classes	60	−7	13	2.50	2.97	0.565	0.161	0.309	−0.0352	0.608
Δ Visual working memory	No extra classes	60	−10	17	6.00	5.43	0.731	−0.271	0.309	−0.148	0.608
	Extra classes	58	−6	24	5.00	7.07	1.01	0.717	0.314	−0.0428	0.618
Δ Cognitive flexibility	No extra classes	64	−5	7	3.00	2.45	0.321	−0.692	0.299	0.275	0.590
	Extra classes	60	−4	9	3.00	2.72	0.424	0.0729	0.309	−0.632	0.608

**Table 3:** Mann–Whitney U test for differences between mean rank of group taking attending extra preschool education center and group not taking any extra classes.

Executive functions component	Age, and Δ	No extra classes, *n* = 64		Extra classes, *n* = 60		U-Mann–Whitney test	*p* level
		M	SD	M	SD		
Inhibition	5–6 y. o.	10.04	3.00	10.65	3.07	1236.000	0.128
	6–7 y. o.	10.61	3.46	10.87	3.88	1340.000	0.377
	Δ	0.57	4.18	0.22	3.13	1369.500	0.482
Physical inhibition	5–6 y. o.	27.35	2.44	27.10	2.28	856.000	0.348
	6–7 y. o.	27.74	3.47	26.67	3.54	782.000	0.116
	Δ	0.39	3.95	−0.43	3.88	853.000	0.342
Verbal working memory	5–6 y. o.	15.90	4.35	20.13	3.41	781.000	0.000
	6–7 y. o.	17.29	5.28	23.10	3.81	703.000	0.000
	Δ	1.39	4.43	2.97	4.38	1433.500	0.028
Visual working memory	5–6 y. o.	33.41	6.38	36.28	3.86	1281.500	0.013
	6–7 y. o.	40.48	5.83	41.68	5.19	1528.500	0.318
	Δ	7.07	7.66	5.43	5.66	1651.500	0.633
Cognitive flexibility	5–6 y. o.	18.47	1.94	17.92	1.62	1624.000	0.133
	6–7 y. o.	20.92	2.67	20.63	2.95	1838.000	0.679
	Δ	2.45	2.57	2.58	2.93	1881.000	0.844

## 4. Discussion

The study aimed to investigate the difference in EF development within a year between children attending the extra preschool education center two times a week for 4 h and children who did not take extra classes. The main hypothesis of the research suggested that children attending extra classes had a greater development rate of EF within a year, compared to children taking no extra classes. This hypothesis is confirmed in part. In the study, there is a difference between the groups in the rate of development of verbal working memory.

There is a difference between the groups not only in the rate of development of verbal working memory but also in the level of verbal working memory at the first EF assessment (T1). Children attending the extra preschool education center initially had a higher level of verbal working memory. The difference in the initial level can be explained by the fact that only children with a high enough level of cognitive development enroll in the educational program at the center. It is shown that verbal working memory is positively associated with fluid intelligence and attentional processes (Engle, 2018; Sala and Gobet, 2020). While children with not mature enough verbal working memory, fluid intelligence, and attentional processes are not enrolled in 4-h classes (Sala and Gobet, 2020).

The difference between the groups in the development rate of verbal working memory can be explained by the features of the classes in the extra preschool education center. Since 5–7 year old children do not yet know how to read quickly, teachers give all children instructions verbally during classes. That means that the children from “extra classes” group two times a week for 4 h actively listened to what a teacher was telling them and operated on this information. Such a process stimulates the development of verbal working memory (Engle, 2018). It is important to note that in the present study, working memory was assessed using the subtest “Sentence Repetition.” This tool evaluates the same processes that children use when listening to instructions – children need to keep exactly and in detail in verbal working memory what the teacher says.

Other factors in addition to extra classes may have influenced the difference between the groups on the topic of growth of verbal working memory. Therefore, the screen time was higher in the “no extra classes” group, and the level of maternal education was lower. Both these factors are associated with the development of EF (Linebarger et al., 2014; Diamond and Ling, 2016; McNeill et al., 2019). However, the absence of differences between the groups in the development rate of other components of EF except verbal working memory suggests that the difference in the development rate of verbal working memory is related precisely to the extra class attendance. Because both screen time and maternal education level are associated with the development of all components of EF, not just verbal working memory (Linebarger et al., 2014; Diamond and Ling, 2016; McNeill et al., 2019).

There are no differences between the groups in the rates of development of cognitive flexibility, visual working memory, cognitive, and physical inhibitory control. One can assume that the absence of differences in these components can be explained by the fact that all children initially had normal EF level, almost all children were from middle and high socioeconomic status families. Although extra classes give a more significant effect for children with an initially low EF level and children from low socioeconomic status families (Diamond and Ling, 2016; Zysset et al., 2018). Also, at preschool age, extra classes stimulate EF development more strongly in boys than in girls, since girls, on average, have a higher EF level at this age (Zysset et al., 2018). And in the current study, the majority of the participants were girls.

The absence of differences in all EF components except verbal working memory can be explained by the fact that the children in both groups attended in kindergarten classes every day. The kindergarten classes last for several hours a day, and the rest of the time in kindergarten children spend on play, which also contributes to the development of EF (Yogman et al., 2018; Rosas et al., 2019). That is, the children in both groups are already in an environment that is favorable for the development of EF. And perhaps the effect of extra classes twice a week is not so great compared to the influences of children’s everyday kindergarten environment.

Another explanation for the absence of difference between the groups is related to the role of parents. Parents can involve children who do not take additional classes in special developmental activities at home. And vice versa, those who take additional classes may not be also engaged at home. Most likely, special developmental activities at home are less disciplined then in extra preschool education center, so there are no situations when a parent gives a lot of instructions to a child. One can assume that in this regard there are differences between the groups in verbal working memory development rate, but there are no differences in other EF components development rate. These other components are probably stimulated in both groups, one group at home and the other in the classroom.

There are many limitations of the study. First, the level of maternal education and screen time are different in the groups. Second, there is no information about classes performance characteristics and about the level of motivation of extras classes participation in the extra classes group. Third, several important factors that may influence EF development were not controlled: quality of parent–child interaction, characteristics of the learning environment in kindergarten and at home, level of physical activity.

## 5. Conclusion

This exploratory study allows one to draw recommendations for further research design. Further longitudinal study should continue more than 1 year. Further research should include different groups of participants: typically developing children and children with special needs, children from families with low socioeconomic status. Also, it is important to include children with an initial low, medium and high EF level and include 50% of boys. It is also crucial to monitor as much as possible factors that may affect EF development. Children taking extra classes should be under observation throughout the whole study period to evaluate the performance characteristics and motivation and attendance frequency. Moreover, to explore which type of extra classes is most effective for EF development, one should compare different training programs and classes with different duration. Also, it is possible to use other methods of EF evaluation to compare sensitivity of the subtests.

## Data availability statement

The raw data supporting the conclusions of this article will be made available by the authors, without undue reservation.

## Ethics statement

The studies involving human participants were reviewed and approved by Ethics Committee of the Lomonosov Moscow State University (approval no: 2022/23). Written informed consent to participate in this study was provided by the participants’ legal guardian/next of kin.

## Author contributions

LB and AD contributed to conception and design of the study. EC gathered and analyzed the data and acquired resources. LB, EC, and AD drafted the manuscript. All authors contributed to the article and approved the submitted version.

## Funding

This work was supported by the Russian Science Foundation Grant No. 21-78-10153.

## Conflict of interest

The authors declare that the research was conducted in the absence of any commercial or financial relationships that could be construed as a potential conflict of interest.

## Publisher’s note

All claims expressed in this article are solely those of the authors and do not necessarily represent those of their affiliated organizations, or those of the publisher, the editors and the reviewers. Any product that may be evaluated in this article, or claim that may be made by its manufacturer, is not guaranteed or endorsed by the publisher.
